# Evaluation of a risk score to predict future *Clostridium difficile* disease using UK primary care and hospital data in Clinical Practice Research Datalink

**DOI:** 10.1080/21645515.2019.1589288

**Published:** 2019-04-04

**Authors:** Clare Marley, Yassine El Hahi, Germano Ferreira, Laura Woods, Ana Ramirez Villaescusa

**Affiliations:** a GSK, Brentford, UK; b Valesta c/o GSK, Mechelen, Belgium; c P95, Heverlee, Belgium; d Department of Non communicable disease epidemiology, London School of Hygiene & Tropical medicine, London, UK; e GSK, Wavre, Belgium

**Keywords:** Clinical Practice Research Datalink, *Clostridium difficile*, Hospital Episode Statistics, nosocomial infection, risk index, hospital-acquired infection, community-acquired infection

## Abstract

We evaluated the applicability of a *Clostridium difficile* infection (CDI) risk index developed for patients at hospital discharge to identify persons at high-risk of CDI in a primary care population. This retrospective observational study used data from the UK Clinical Practice Research Datalink, linked with Hospital Episodes Statistics. The risk index was based on the following patient characteristics: age, previous hospitalizations, days in hospital, and prior antibiotics use. Individual risk scores were calculated by summing points assigned to pre-defined categories for each characteristic. We assessed the association of risk factors with CDI by multivariate logistic regression. The estimated CDI incidence rate was 4/10,000 and 2/10,000 person-years in 2008 and 2012, respectively. On an index with a maximal risk of 19, a cut-off for high risk of ≥7 had sensitivity, specificity and positive predictive values of 80%, 87% and 12%, respectively. A high-risk person had a ~ 35% higher risk of CDI than a low-risk person. Multivariate risk factor analysis indicated a need to reconsider the relative risk scores. The CDI risk index can be applied to the UK primary care population and help identify study populations for vaccine development studies. Reassessing the relative weights assigned to risk factors could improve the index performance in this setting.

## Introduction

*Clostridium difficile* is an anaerobic spore forming bacterium, which is commonly found in the environment and transmitted among humans through the fecal-oral route.^1,2^ Approximately 3% of healthy adults and up to 66% of infants have *C. difficile* in their gut without it causing any harm. However, it can cause disease when the normal commensal flora is reduced or absent, for example due to antibiotic exposure.^1^ *C. difficile* cells cause disease by the release of different toxins which can damage the intestinal mucosa. Symptoms include diarrhea of varying severity, abdominal pain, fever, mild to severe and even life-threatening inflammation of the bowel (pseudomembranous colitis).^1,3^

*C. difficile* infection has traditionally been considered as mainly a hospital- or other healthcare facility-acquired infection, but since the 1990s community-acquired *C. difficile* infection reports have increased. In the United States (US), about 25% of all *C. difficile* cases in 2011 were reported as community-acquired.^4^ A recent review showed that the incidence of community-acquired infection has continued to increase over the last decade, accounting for up to 41% of all *C. difficile*-related cases.^5^

The US Centers for Disease Control and Prevention (CDC) has created a risk index to identify hospitalized patients at high risk of developing *C. difficile* infection after hospital discharge based on readily available data.^6^ One of the aims of generating this risk index was to identify groups of people at high risk of infections with *C. difficile* for recruitment to clinical trials for the development of vaccines.^6^ Currently there is no vaccine available against *C. difficile*. Identifying a target population for future clinical trials in support of vaccine development is essential.

In the present study, we examined the feasibility of applying the CDC risk index to data originating in primary care settings across the United Kingdom (UK) and its ability to identify groups at high risk of *C. difficile* infection in a general, unselected population of adults aged ≥18 years old. We also assessed the predictive value of potential risk factors for *C. difficile* infection and their relative importance when adjusting for the impact of other potential risk factors.

Widely recognized risk factors are hospitalization, advanced age and prior antibiotics use.^7^ However, the causes of the changing epidemiology and the impact of possible new risk factors are yet to be elucidated.^2^ Several studies have been published identifying risk factors for *C. difficile* infection and developing prediction rules with the aim to improve management of individuals deemed to be at the highest risk.^6,8–25^ Most of these studies target patients admitted to hospitals or living in long-term healthcare facilities.^9–11,17–19,22–24,26–28^ or *C. difficile* infection cases risking recurrence.^13,19,21,25^

The present study adopts a novel approach by targeting a general, unselected population of adults in the primary care setting coupled with secondary care data for the same population to aim for a comprehensive detection of cases of *C. difficile* infection.

## Results

### Overall incidence of C. difficile infection

**Figure 1.**
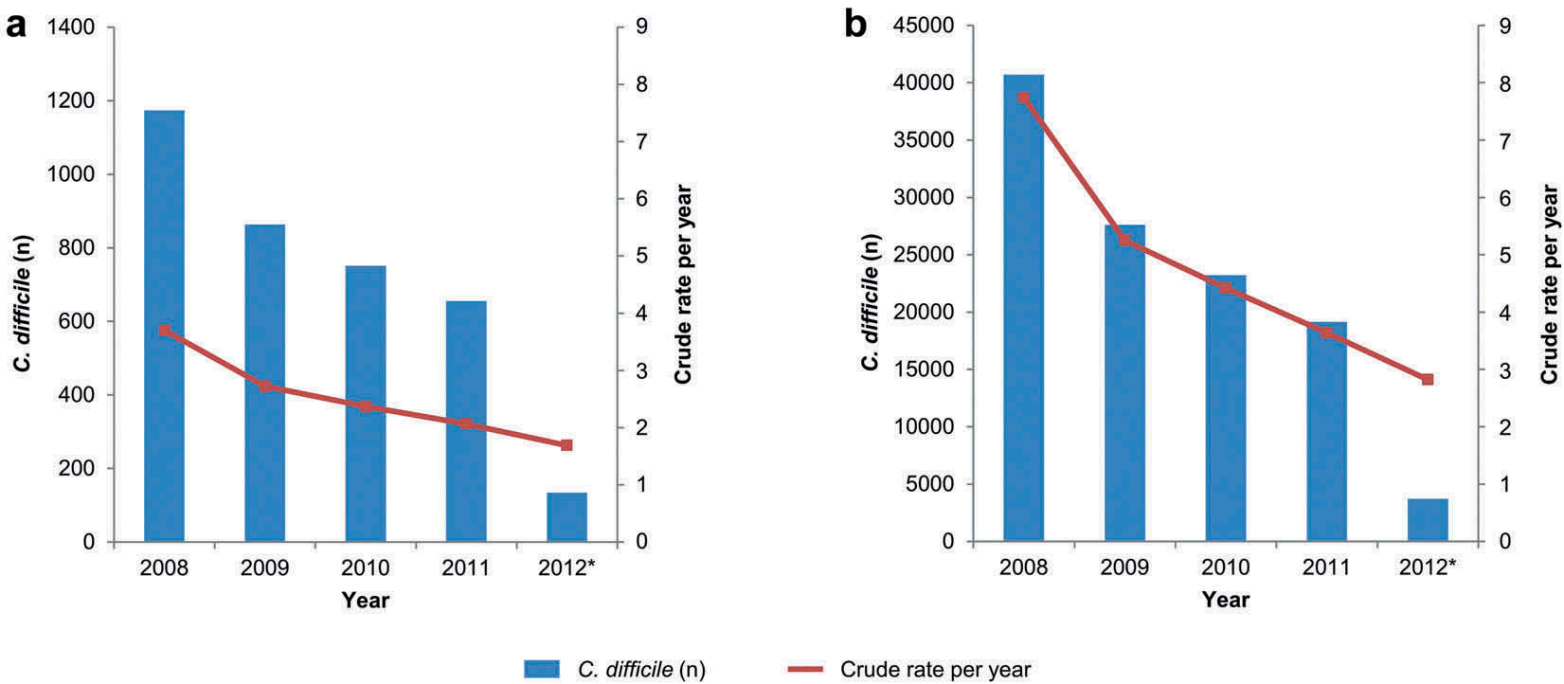
**All *C. difficile* infection cases and the crude annual incidence rates per 10,000 person-years for CPRD/HES (A) and PHE (B)** n: number of cases, CPRD: Clinical Practice Research Datalink database, HES: Hospital Episode Statistics database, PHE: Public Health England database * 2012 contains data from 1st January – 31st of March only. The crude rate is based on estimated full year data (2012 figures were multiplied by 4 to make full year estimates).

Data were extracted from the Clinical Practice Research Datalink (CPRD) GOLD database, which is linked to secondary care datasets, including the Hospital Episodes Statistics (HES), and contains details of all hospital admissions (admitted patient care [APC]), outpatient visits, and emergency unit attendances at National Health Service (NHS) hospitals in the UK. Between January 2008 and March 2012, 3,576 *C. difficile* infection cases were identified in the CPRD/HES population subjects, including all those aged 2 years of age or older and 114,395 *C. difficile* infection cases recorded by the PHE surveillance system in the entire English population. The annual incidence rate in the CPRD/HES dataset was 4/10,000 person years (PY) in 2008 and decreased from year to year to 2/10,000 PY in 2012 (Figure 1A). Public Health England (PHE, formerly the Health Protection Agency) data also showed a declining trend from 8/10,000 PY in 2008 to 3/10,000 PY in 2012 (Figure 1B).

### Demographics and risk factor characteristics

Table 1 compares the CPRD/HES study population with the US population for which the *C. difficile* infection risk index was developed, in terms of the co-variates (and categories) on which the CDC risk index is based. The CPRD/HES adult study population included 2,492,493 individuals aged ≥18 years. The age and sex distribution were similar in the 2 populations. Prior to the inpatient hospital stay, at the end of which the US study population was recruited at discharge, the vast majority had not been hospitalized; almost all the individuals in the CPRD/HES study population had no inpatient hospital stay during the risk assessment period. With regards to antibiotic use, 7% of the CPRD/HES study population had used antibiotics in the 90 days before the index date, whereas 45% of the CDC patients had used antibiotics during the corresponding time period.

**Table 1.** Demographics for the CPRD/HES and CDC study populations.

Characteristic	CPRD/HES population N (%)	CDC population* N (%)
**Study population size**	2,492,493 (100)	35,186 (100)
**Sex**		
Men	1,200,764 (48.18)	13,917 (39.55)
Women	1,291,729 (51.82)	21,269 (60.45)
**Age (years)**		
18–39	876,217 (35.15)	11,715 (33.29)
40–49	477,103 (19.14)	4,941 (14.04)
50–64	597,993 (23.99)	8,260 (23.48)
65–74	275,752 (11.06)	4,253 (12.09)
75+	265,428 (10.65)	6,017 (17.10)
**Number of hospitalizations in previous 90 days**		
0	2,465,563 (98.92)	31,267 (88.86)
1	23,324 (0.94)	3,020 (8.58)
2+	3,606 (0.14)	899 (2.55)
**Length of stay (days)**		
0	2,465,578 (98.92)	N/A
1–3	15,888 (0.64)	21,381(60.77)
4–9	5,422 (0.22)	10,394 (29.54)
10+	5,605 (0.22)	3,411 (9.69)
**Number of antibiotic classes used in previous 90 days**		
0	2,328,598 (93.42)	19,208 (54.59)
1	150,351 (6.03)	9,102 (25.87)
2	12,580 (0.50)	4,116 (11.70)
3	910 (0.04)	1,782 (5.06)
4	51 (0.00)	676 (1.92)
5+	3 (0.00)	302 (0.86)

*Data from Baggs et al.^6^

N: number of persons, %: percentage of persons in this category out of the total study population, CPRD: Clinical Practice Research Datalink database, HES: Hospital Episode Statistics database, CDC: US Centers for Disease Control and Prevention.

### Risk classification of the study population

**Figure 2.**
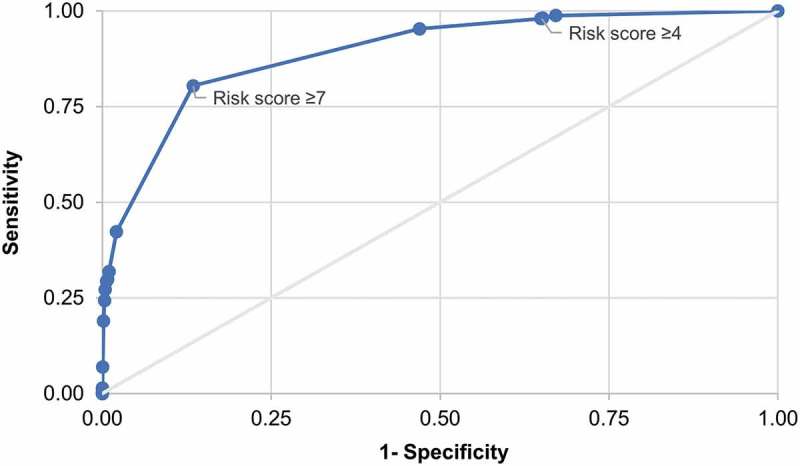
Receiver operating characteristic curve for varying risk scores for *C. difficile* infection within 90 days. Note: Area under the receiver operating characteristic curve = 0.8926.

The receiver operating characteristics (ROC) curve presented in Figure 2 shows the sensitivity and 1-specificity of the risk index for *C. difficile* infection within 90 days of the index date for varying cut-off values. An analysis by risk score cut-off value was attempted informally to optimize the selection of the cut-off risk score by assessing the trade-off between sensitivity and specificity. The highest of the calculated risk scores was 19. Details of the specificity, sensitivity, positive likelihood ratio (LR+) and the percentage of subjects correctly classified for varying cut-off values of the risk score are given in **Supplementary Material, Table SM1**.

Applying the CDC risk score cut-off value of 4 used by Baggs et al.,^6^ the odds ratio (OR) of developing *C. difficile* infection within 90 days of the index date was 26 times higher in high-risk than in low-risk individuals (OR = 25.79, 95% confidence interval [CI]: 13.79–48.24). With the cut-off value of 4, the sensitivity of the risk index was 98% and the specificity was 35%. Thirty-five percent of cases were correctly classified and the LR+ was 1.51. **Table SM1** presents the sensitivity, specificity, proportion of correctly classified and LR+ for all possible values of the cut-off.

Informal, visual inspection of the ROC curve in Figure 2 suggests that a cut-off value of 7 could result in optimization of the trade-off between sensitivity and specificity. Thus, defining high risk as a score ≥7, the sensitivity was 80% and specificity 87%; the positive predictive value (PPV) was 0.12%, the LR+ increased to 5.98 and 87% of *C. difficile* infection cases were being correctly classified. The summary statistics indicating the discriminatory capacity of the risk index at cut-off values of 4 and 7, respectively, are summarized in Table 2, which includes the summary statistics for *C. difficile* infection cases with onset within 365 days of the index date in addition to those with onset within 90 days.

**Table 2.** Risk classification and *C. difficile* infection within 90 days or 365 days of index date with cut–off risk scores set to either 4 or 7 (sensitivity analysis).

	Cut–off value = 4				Cut–off value = 7			
	90 days		365 days		90 days		365 days	
	Case	Non–case	Case	Non–case	Case	Non–case	Case	Non–case
High–risk	480	1,621,077	1,290	1,620,267	394	335,023	983	334,434
Low–risk	10	870,926	42	870,894	96	2,156,980	349	2,156,727
Total	490	2,492,003	1,332	2,491,162	490	2,492,003	1,332	2,491,161
OR (95% CI)	25.79 (13.79–48.24)		16.51 (12.14–22.45)		26.42 (21.14–33.03)		18.16 (16.08–20.52)	
PPV	0.03		0.08		0.12		0.29	
NPV	99.99		99.99		100		99.98	
LR+	1.51		1.49		5.98		5.50	

High–risk: persons with a risk score ≥ 4 (cut–off value = 4) or ≥ 7 (cut–off value = 7); Low–risk: persons with a risk score < 4 (cut–off value = 4) or < 7 (cut–off value = 7); OR: odds ratio; CI: confidence interval; PPV: positive predictive value; NPV: negative predictive value; LR+: positive likelihood ratio.

The cut-off risk score of 7 categorized 2,157,076 (86.5%) individuals as low-risk and 335,417 (13.5%) as high-risk (Table 3). Altogether, 490 cases of *C. difficile* infection occurred within 90 days of the index date, 96 (19.6%) in individuals classified as low-risk and 394 (80.4%) in those identified as high-risk.

**Table 3.** The study population by risk status and *C. difficile* infection status within 90 days of the index date.

	*C. difficile* infection (90 days)			
	Low–risk (risk score <7)		High–risk (risk score ≥7)	
Variable	Case (N = 96) n (%)	Non–case (N = 2,156,980) n (%)	Case (N = 394) n (%)	Non–case (N = 335,023) n (%)
**Sex**				
Men	46 (47.92)	1,065,758 (49.41)	166 (42.13)	134,794 (40.23)
Women	50 (52.08)	1,091,222 (50.59)	228 (57.87)	200,229 (59,77)
**Age (years)**				
18–39	12 (12.50)	875,769 (40.60)	5 (1.27)	431 (0.13)
40–49	13 (13.54)	472,429 (21.90)	5 (1.27)	4,656 (1.39)
50–64	33 (34.38)	557,364 (25.84)	22 (5.58)	40,574 (12.11)
65–74	38 (39.58)	251,418 (11.66)	36 (9.14)	24,260 (7.24)
75+	0 (0.00)	0 (0.00)	326 (82.74)	265,102 (79.13)
**Previous hospitalization**				
0	94 (97.92)	2,152,144 (99.78)	244 (61.93)	313,081 (93.45)
1	1 (1.04)	4,435 (0.21)	106 (26.90)	18,782 (5.61)
2+	1 (1.04)	401 (0.02)	44 (11.17)	3,160 (0.94)
**Length of stay (days)**				
**0**	94 (97.92)	2,152,147 (99.78)	244 (61.93)	313,093 (93.45)
1–3	2 (2.08)	4,228 (0.20)	21 (5.33)	11,637 (3.47)
4–9	0 (0.00)	605 (0.03)	20 (5.08)	4,797 (1.43)
10+	0 (0.00)	0 (0.00)	109 (27.66)	5,496 (1.64)
**Previous antibiotics used (number of classes)**				
0	89 (92.71)	2,080,266 (96.44)	286 (72.59)	247,957 (74.01)
1	6 (6.25)	73,556 (3.41)	88 (22.34)	76,701 (22.89)
2	1 (1.04)	3,031 (0.14)	16 (4.06)	9,532 (2.85)
3	0 (0.00)	120 (0.01)	3 (0.76)	787 (0.23)
4	0 (0.00)	7 (0.01)	1 (0.25)	43 (0.01)
5+	0 (0.00)	0 (0.00)	0 (0.00)	3 (0.00)

N: total number of cases or non–cases in this risk category; n: number of cases or non–cases in this category of the variable; %: (n/N) x100.

Antibiotics classes considered for inclusion: Aminoglycosides, 1st to 4th generation cephalosporins, fluroquinolones, vancomycin IV, betalatamase, penicillins, clindamycin, macorlides, sulfanomides antibiotics, carbapenhems, other antibiotics.

In the low-risk group, the overall proportion with *C. difficile* infection was 0.0045% and the proportion was similar in men and women. The proportion with *C. difficile* infection increased 10-fold with age, from 0.0014% in the 18–39-year-old group to 0.015% in those aged 65–74 years. There were no individuals aged ≥75 years with *C. difficile* infection.

In the high-risk group, the overall proportion with *C. difficile* infection was 0.12%, similar for men and women. The proportion was between 8 and 10 times higher in the age group 18–39 years than in the older age groups. For individuals with previous hospitalization, the overall proportion with *C. difficile* infection was 0.68%; those with 2 or more hospital stays were infected more than twice as often as those with 1 hospital stay, 1.37% versus 0.56%. The proportion of infected individuals also increased with the length of hospital stays to 1.98% for those with stays of ≥10 days, which is 10-fold higher than for those with stays of 1–3 days. Among individuals having used antibiotics prior to the index date, the proportion with *C. difficile* infection increased from 0.12% among those having used 1 class to 0.38% among those having used 3 classes.

### Logistic regression analysis of risk factors

The 3,526 cases of *C. difficile* infection in individuals aged ≥18 years were included in univariate and multivariate logistic regression models to determine the relative weight of the potential risk factor categories. In the univariate analyses, all categories of each risk factor were found to be statistically significant at the p = 0.05 level and were thus retained in the multivariate analysis. However, there was a high degree of collinearity between the number of previous hospitalizations and the total number of days spent in hospital, so the latter variable was left out of the multivariate analysis.

When controlling for the other risk factors, the strongest associations with developing *C. difficile* infection were found for having been hospitalized twice (OR = 115.36, 95% CI: 104.36–127.51), having been treated with 5 or more classes of antibiotics (OR = 70.31, 95% CI: 2.55–1934.91) and having been hospitalized once (OR = 47.76, 95% CI: 43.84–52.03) (Table 4).

**Table 4.** Logistic regression for assessment of the association of risk factors assessed during the 90 days before *C. difficile* infection onset.

	CDI		Univariate analyses	Multivariate analysis
Variable	Case (N = 3,526) n (%)	Non–case (N = 2,488,967) n (%)	OR (95% CI)	Adjusted OR* (95% CI)
**Age (years)**				
18–39	118 (3.35)	825,446 (33.16)	Reference category	Reference category
40–49	118 (3.35)	472,152 (18.97)	1.75 (1.35–2.26)	1.67 (1.29–2.16)
50–59	417 (11.83)	600,870 (24.14)	4.85 (3.96–5.96)	3.84 (3.12–4.71)
60–74	614 (17.41)	294,380 (11.83)	14.59 (11.98–17.77)	7.96 (6.53–9.72)
75+	2,259 (64.07)	296,119 (11.90)	53.37 (44.35–64.22)	15.69 (13.00–18.95)
**Hospitalized before**				
0	878 (24.90)	2,431,332 (97.68)	Reference category	Reference category
1	1,736 (49.23)	48,703 (1.96)	98.70 (90.96–107.10)	47.76 (43.84–52.03)
2+	912 (25.87)	8,932 (0.36)	282.74 (257.13–310.91)	115.36 (104.36–127.51)
**Length of stay (days)**				
0	892 (25.30)	2,431,411 (97.69)	0.09 (0.08–0.11)	
1–3	125 (3.55)	31,460 (1.26)	Reference category	Not included in the adjusted model
4–9	313 (8.88)	12,533 (0.50)	6.28 (5.10–7.74)	
10+	2,196 (62.28)	13,563 (0.54)	40.75 (33.99–48.85)	
**No. of antibiotics classes used**				
0	2,348 (66.59)	2,278,820 (91.56)	Reference category	Reference category
1	897 (25.44)	188,347 (7.57)	4.62 (4.28–4.99)	2.07 (1.91–2.25)
2	241 (6.83)	20,203 (0.81)	11.58 (10.13–13.23)	3.34 (2.89–3.85)
3	35 (0.99)	1,508 (0.06)	22.53 (16.07–31.57)	4.94 (3.39–7.20)
4	4 (0.11)	85 (0.00)	45.67 (16.74–124.60)	7.30 (2.31–23.04)
5+	1 (0.03)	4 (0.00)	242.81 (27.14–2172.16)	70.31 (2.55–1934.91)

CDI: *Clostridium difficile* infection; OR: odds ratio; CI: confidence interval; N: number of cases or non–cases; n: number of cases (or non–cases) in this category; %: (n/N)x100.

Antibiotics classes considered for inclusion: Aminoglycosides, 1st to 4th generation cephalosporins, fluroquinolones, vancomycin IV, betalatamase, penicillins, clindamycin, macorlides, sulfanomides antibiotics, carbapenhems, other antibiotics.

## Discussion

This study was the first to use the CPRD-linked HES APC database to estimate the incidence of *C. difficile* infection in a primary care setting. Over the 4-year study period, from January 2008 to March 2012, the incidence of *C. difficile* infection was found to fall steadily, with an overall decrease of the annual incidence rate per 10,000 PY of about 50%. A similar, although even more pronounced, decrease in the overall annual incidence rate per 10,000 PY over the same period was observed in the national surveillance data recorded by PHE. The validation against the PHE data reinforces the feasibility of measuring risk factors for *C. difficile* infection in the CPRD/HES data. The higher number of *C. difficile* infection cases reported in 2008 in the PHE database may be linked to the epidemic of Toxin B strains of *C. difficile* infection that happened at that time in Europe.^29^

Applying the CDC risk index to the CPRD/HES population to assess its performance and usefulness in the primary care context showed that the CDC cut-off value of the risk score of 4 gave a very low PPV and also a LR+ value close to 1. Selecting 7 as an alternative cut-off value of the risk score based on visual inspection of the ROC curve and detailed analyses by risk scores, resulted in relatively high sensitivity and specificity, correct classification of 87% rather than 35% of the individuals, and a LR+ value of 5.98. An LR+ value of this order suggests an increased risk for *C. difficile* infection of about 35% for the high-risk group compared to the low-risk group (cf. the examples provided by McGee in his introduction of the simplified LR+ measure^30^).

With 4 as the cut-off value for the risk score, 2 thirds of our study population were in the high-risk group. The cut-off value of 7 seems to be more appropriate for clinical application in primary care for a number of reasons. Both the sensitivity and specificity are higher, meaning that the test is more effective at distinguishing between individuals at risk and not at risk. It also means that risk factors beyond age alone are contributing to an individual’s risk status, whereas the cut-off value of 4 places all persons aged ≥40 in the high-risk group. The cut-off of 7 also augmented the PPV to 12% rather than 3%.

Multivariate logistic regression analysis of the risk factors indicated that the factors with the highest independent association with developing *C. difficile* infection, when controlling for the other factors, were: having been hospitalized during the 90 days preceding the *C. difficile* infection episode; having used 4 or more classes of antibiotics over the same period; and being 75 years old or older. The relative scores assigned to the risk factor categories may in future development of the risk index be better adapted to a general primary care population by using the OR estimates derived in the multivariate risk factor analysis.

For comparison with other risk indices for *C. difficile* infection used in previous studies, it must first be noted that only few of these have focused on primary care settings. In a recent study using a Medicare 5% random sample, representative of the US population >65 years of age, high-risk individuals were identified with the ‘population attributable risk percentage’ (PAR%).^12^ The PAR% combines determination of the independent importance of individual risk factors with an assessment of the prevalence of each risk factor in the targeted population.^12^ An exploratory examination of the applicability of this notion to the results of our study indicates that the PAR% associated with the risk factor with the highest OR in the multivariate risk factor analysis would result in a low PAR%, as only 0.22% of the population had 2 or more hospitalizations.

Limitations of the study include the fact that the HES APC data do not record medications used during hospital admissions. Consequently, the use of antibiotics may have been underestimated for the 1.6% of the population that had been hospitalized at least once before the risk assessment unless the use had been recorded in the general practitioner (GP)’s notes or the GP prescribed continued antibiotics use after discharge of the patient. The study must also be seen in the light of general limitations of CPRD data,^31^ including variations in data quality due to the data being recorded during routine GP consultations, rather than for research purposes.

One strength of the study was the large number of individuals observed, meaning that very precise estimates could be obtained and that no imputations were made for missing values. Furthermore, since the risk factors examined are readily available data, no additional data collection was required for the use of the risk index to identify individuals at elevated risk for *C. difficile* infection.

## Conclusions

Each of the variables included in the risk score was significantly associated with the risk of *C. difficile* infection (p-value <0.05). The risk factors identified in this study are well recognized and reported in the literature and our study confirmed that it is possible to measure them in the CPRD/HES databases, with the proviso that antibiotics use during hospitalizations may not be captured.

The performance of the CDC score in the primary care sector using CPRD/HES data was low. Assigning a higher risk score as cut-off value between low and high risk based on optimization of the trade-off between sensitivity and specificity improved the performance of the risk index considerably. However, further refinement beyond adjustment of the cut-off value is required to increase its usefulness in a primary care setting. Given the variables now included, a possible improvement would be to adapt the relative weights assigned to the individual categories of the risk factors. The inclusion of other readily available variables previously identified as risk factors for *C. difficile* infection (such as underlying immunosuppression, renal insufficiency or history of diabetes, or use of proton pump inhibitors) could also be considered.

As the overall prevalence of *C. difficile* infection is low, the PPV of any predictive tool remains moderate, meaning that most individuals classified as high-risk will not develop *C. difficile* infection. Nevertheless, such tools may be relevant and useful if they are convenient and inexpensive, especially if they have a negative predictive value around 100%. The results can optimize the identification of patients to be targeted for individual *C. difficile* testing and epidemiological assessment and potentially, the identification of antibiotic resistant strains. In addition, they can also be used to better identify target populations for inclusion in clinical trials for vaccine development and other preventive interventions against *C. difficile* infection.

## Methods

This retrospective observational study used data extracted from the CPRD. The CRPD includes anonymized electronic records from GPs across the UK. The database contains records including medical diagnoses, referrals to secondary care, prescriptions, diagnostic test results and all other types of care administered as part of GP routine work. The CPRD GOLD database, linked to the HES dataset, was used for this study. As of January 2014, the CPRD contained records of more than 13 million persons of whom approximately 5.5 million were still active (alive, currently registered), representing about 8.5% of the UK population during the study period. The HES data link was available for approximately 60% of the individuals in the CPRD database.^32^ CPRD is broadly representative of the characteristics of patients (in terms of age, gender and ethnicity) and GP practices in the UK.^31,33^

The index date for the start of follow up for an individual was the date of the first record of a *C. difficile* infection such as his/her first GP visit or the first prescription during the study period, to ensure that the individual was active in the cohort. Individuals with a history of *C. difficile* infection or exposure to related treatments (i.e., vancomycin, metronidazole, fidaxomicin) during the 90 days prior to the index date were excluded. The risk assessment was made at the index date for each participant.

To identify *C. difficile* infection cases, the PHE standard case definition^3^ was used. Since 2007, all NHS trusts in England have been requested to report all cases of *C. difficile* infection using this definition in individuals aged 2 years or older as part of a program of mandatory surveillance of *C. difficile* infection initiated in 2004. Based on this definition, a list of Read codes was established and used for data extraction from the CPRD/HES dataset.

An analysis of the overall incidence of *C. difficile* infection was performed to assess the capture of *C. difficile* infection cases in the CPRD/HES dataset compared to that of the mandatory PHE surveillance.^3^ For this analysis, individuals aged 2 years or older were included in the cohort, provided they met the standard CPRD requirements for acceptability for research,^31^ were eligible for the HES link, and had been included in the database for at least 12 months at the time of inclusion in the study cohort.

Overall annual incidence rates were calculated for the CPRD/HES cohort and compared to the incidence rates reported by PHE as a validation of the CPRD/HES dataset. The incidence was expressed as the annual incidence rate per 10,000 PY. For 2012, the annual rate was estimated by extrapolation of the number of cases observed until the end of March.

For the main analysis of the validity of the CDC risk index and the assessment of the relative importance of the potential risk factors for *C. difficile* infection, only individuals aged 18 years or older were included in the study cohort.

The CDC risk index is based on the following co-variates with respective categories: age (18–39, 40–49, 50–64, 65–74, and 75+), previous hospitalizations (0, 1, 2+), length of stay in hospital (0, 1–3 days, 4–9, 10+), and use of antibiotics (0, 1 class, 2 classes, 3, 4, 5 classes). Previous hospitalizations, length of stay in hospital, and use of antibiotics classes other than *C. difficile*-related antibiotics were assessed over the 90 days prior to the index date for the assessment of risk. The reference category for each variable was given a score of 0 and the number of points assigned to the other categories is presented in **Table SM2**.

Further details about the assignment of points to the different categories may be found in the original paper presenting the CDC index in a study of adult residents of Emerging Infection Programs at 2 academic centers; participants were included at discharge after an inpatient stay in one of the study hospitals, conditional on having no history of *C. difficile* infection.^6^

The primary outcome of the validation analysis was the risk score of *C. difficile* infection development ≤90 days or ≤365 days after the risk assessment index date in low-risk and high-risk individuals, respectively. A ROC curve for the trade-off between the sensitivity and specificity of the risk index was generated, and the area under the curve determined.

The performance of the selected index cut-off score in discriminating between high and low-risk individuals was summarized in terms of sensitivity, specificity, PPV, negative predictive value, and its LR+. The latter is a relatively rarely used measure of diagnosing accuracy and it estimates the likelihood of a particular finding in a person with the disease divided by the likelihood of the same finding in a person without the disease.^29^

The predictive value of potential risk factors was assessed by multivariate logistic regression analysis, determining the OR with corresponding CI for each factor. For the *C. difficile* infection cases in these analyses, the presence and ‘grade’ of the risk factors were assessed for the 90 days prior to the date of *C. difficile* infection diagnosis. For cohort participants without *C. difficile* infection, GP consultations following the index date were recorded during the study period. The assessment of risk factors was done for the 90 days period preceding a randomly chosen consultation from the follow-up period.
