# Supplementary material for: Evaluation of a risk score to predict future Clostridium difficile disease using UK primary care and hospital data in Clinical Practice Research Datalink
Source: Hum Vaccin Immunother. 2019 Apr 4;15(10):2475–81. doi: 10.1080/21645515.2019.1589288 (PMC6816380; doi:10.1080/21645515.2019.1589288)
Supplement: Supplemental Material [file khvi-15-10-1589288-s001.docx]

# Supplementary Material

# Table SM1. Sensitivity and specificity by risk score for *C. difficile* infection within 90 days

|  | ***C. difficile* infection within 90 days** | | | |
| --- | --- | --- | --- | --- |
| **Risk score cut–off** | **Sensitivity (%)** | **Specificity (%)** | **Correctly classified (%)** | **LR+** |
| ≥0 | 100 | 0 | 0.02 | 1.00 |
| ≥1 | 98.78 | 32.90 | 32.91 | 1.47 |
| ≥2 | 98.16 | 34.82 | 34.84 | 1.51 |
| ≥3 | 97.96 | 34.94 | 34.96 | 1.51 |
| **≥4** | **97.96** | **34.95** | **34.96** | **1.51** |
| ≥5 | 97.96 | 35.08 | 35.09 | 1.51 |
| ≥6 | 95.31 | 53.06 | 53.07 | 2.03 |
| **≥7** | **80.41** | **86.56** | **86.55** | **5.98** |
| ≥8 | 42.24 | 97.90 | 97.89 | 20.11 |
| ≥9 | 31.84 | 99.00 | 98.99 | 31.87 |
| ≥10 | 29.80 | 99.19 | 99.18 | 36.75 |
| ≥11 | 29.39 | 99.39 | 99.38 | 48.17 |
| ≥12 | 27.14 | 99.56 | 99.55 | 61.79 |
| ≥13 | 24.29 | 99.68 | 99.66 | 75.11 |
| ≥14 | 18.98 | 99.81 | 99.79 | 98.99 |
| ≥15 | 6.94 | 99.94 | 99.92 | 120.49 |
| ≥16 | 1.43 | 99.99 | 99.97 | 141.85 |
| ≥17 | 0.41 | 100.00 | 99.98 | 328.21 |
| ≥18 | 0.00 | 100.00 | 99.98 | – |
| ≥19 | 0.00 | 100.00 | 99.98 | – |
| >19 | 0.00 | 100.00 | 99.98 | – |

%: percentage; LR+: positive likelihood ratio; correctly classified: % of cases correctly classified with this risk score cut–off value.

# Table SM2. *C. difficile* infection risk index

| **Characteristic** | **Points** |
| --- | --- |
| Age 40–49 | 5 |
| Age 50–64 | 6 |
| Age 65–74 | 6 |
| Age 75+ | 7 |
| 1 Past inpatient hospital stay | 4 |
| 2 or more past inpatient hospital stays | 5 |
| Length of Stay (LOS) 1–3 days | 0 |
| LOS 4–9 days | 2 |
| LOS 10+ days | 3 |
| 1 Antibiotic Class | 1 |
| 2 Antibiotic Classes | 2 |
| 3 Antibiotic Classes | 3 |
| 4 Antibiotic Classes | 4 |
| 5+ Antibiotic Classes | 5 |

The *C. difficile* infection risk index developed by the CDC is based on a cohort of hospitalized inpatients in the US. A multivariate Cox proportional hazard regression model identified the most important characteristics associated with *C. difficile* infection. Points for each characteristic identified were assigned by dividing each model parameter estimate by the absolute value of the smallest parameter estimate in the model.

Note: This index was provided by CDC in a previous project report and differs from the 2 indices described in the paper by Baggs et al. (Vaccine. 2015;33(46):6241–9. doi: 10.1016/j.vaccine.2015.09.078 ), not only in the absolute scores but also in the relative weights given to the categories.
